# The high-affinity IgG receptor FcγRI modulates peripheral nerve injury-induced neuropathic pain in rats

**DOI:** 10.1186/s13041-019-0499-3

**Published:** 2019-10-22

**Authors:** Yingxia Liang, Zhiyu Zhang, Zhaodong Juan, Rui Zhang, Can Zhang

**Affiliations:** 1 Department of Anesthesiology, Weifang Medical University, Weifang, 261053 Shandong China; 2 Genetics and Aging Research Unit, McCance Center for Brain Health, Department of Neurology, MassGeneral Institute for Neurodegenerative Diseases (MIND), Massachusetts General Hospital and Harvard Medical School, Charlestown, MA 02129 USA; 3 Department of Trauma Orthopedics, Shouguang People’s Hospital, Weifang, 262700 Shandong China

**Keywords:** Neuropathic pain, Fc gamma receptor, Anti-FcγRI antibody, Spinal cord, Peripheral nerve injury, Inflammatory mediators

## Abstract

The Fc gamma receptor I (FcγRI; CD64) is the high-affinity receptor of the immunoglobulin G protein (IgG). It is usually expressed in immune cells and has recently been identified to distribute in the nervous system and play critical roles in various neurological disorders. Presently, the impacts of FcγRI in neuropathic pain was largely unknown. Here, we aimed to investigate the impacts of FcγRI in neuropathic pain through pain-related neurobehavioral studies and underlying mechanisms by biochemical methods in animal and cell models. Specifically, we first utilized the chronic constriction injury (CCI) rat model that displayed neuropathic pain related symptoms and signs, including thermal hyperalgesia and mechanical allodynia. These neurobehavioral defects were significantly attenuated by the anti-FcγRI antibody, which was associated with reduced levels of neuropeptide substance P, C_3_, and TNF-α. Furthermore, we validated our animal findings using the embryonically neural crest-originated PC12 cell model. We found that stimulation of the IgG immune complex led to increased levels of FcγRI and inflammatory mediators, which were attenuated by the anti-FcγRI antibody in these cells. Collectively, our results from animal and cell-based studies suggest that FcγRI is a critical player for peripheral nerve injury-induced neuropathic pain by mediating pain-related immunological events, which therefore may provide a new therapeutic target for protection against chronic pain.

## Main text

Neuropathic pain, resulting from somatosensory nervous system dysfunction, is characterized by allodynia, hyperalgesia and spontaneous pain [1]. Neuropathic pain is closely related to immunological responses [2–5], which commonly displays elevated levels of antigen-specific immunoglobulins, particularly the presence of immune complexes of IgG and/or IgG in serum [6]. Fc-gamma receptors (FcγRs), the receptors of IgG, were typically expressed on immune cells and may trigger effector responses including cytokine production and phagocytosis [7]. Besides immune cells, IgG and FcγRs also were identified and distributed on neurons of the central and peripheral nervous system [8–10]. Moreover, FcγRs were increasingly recognized for their involvement in various neurological disorders including Alzheimer’s diseases, Parkinson’s disease, ischemic stroke, and multiple sclerosis [11, 12]. The increased knowledge of FcγRs in the nervous system pathophysiology has led to novel preventative and therapeutic strategies for neurological disorders [13]. FcγRI is the high-affinity IgG receptor of the IgG receptor family proteins [14]. In this study, we investigated the effects of FcγRI on neuropathic pain and inflammatory mediators induced by peripheral nerve injury.

**Fig. 1:**
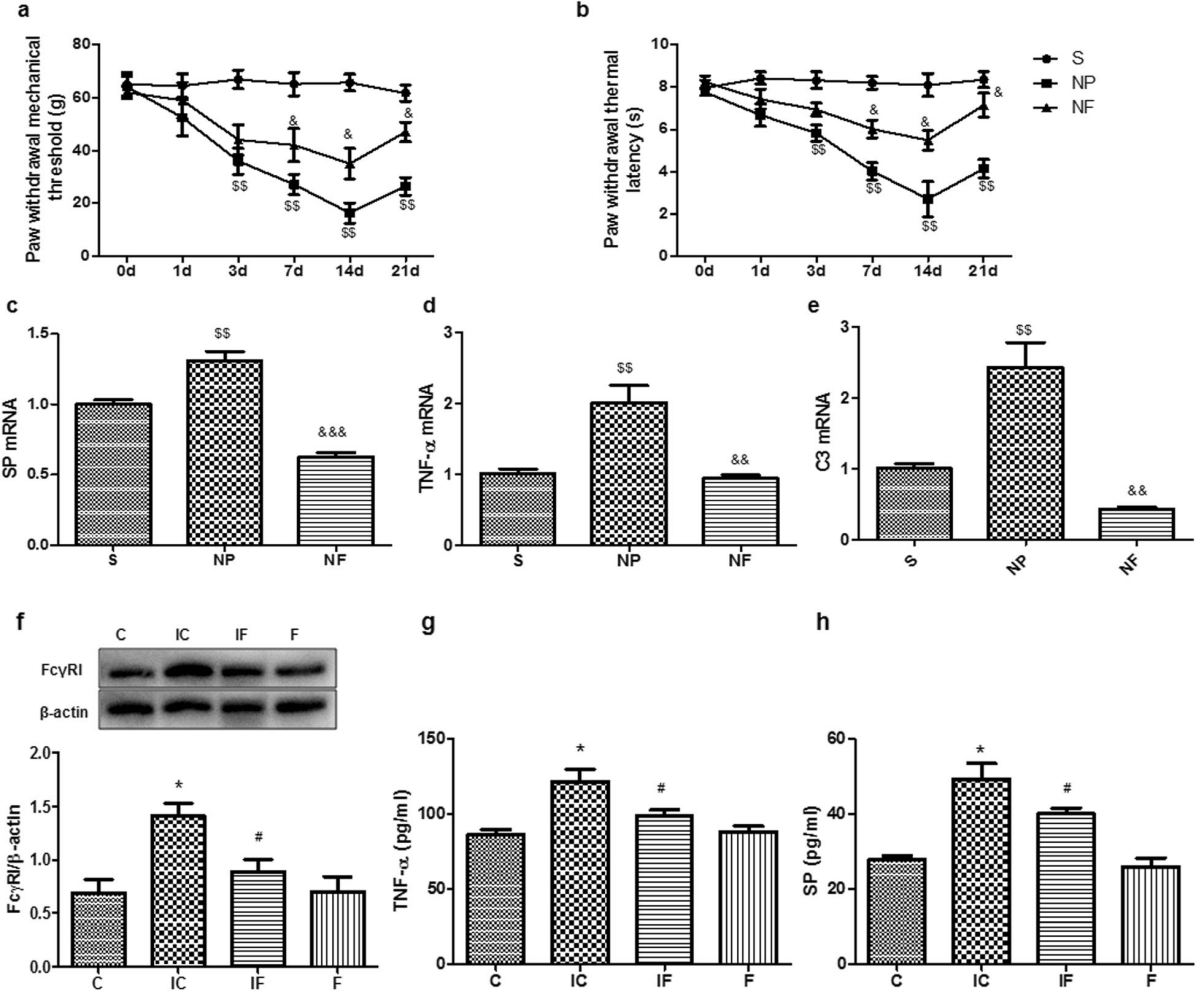
The high-affinity IgG receptor FcγRI modulates peripheral nerve injury-induced neuropathic pain. Modulation of FcγRI by using the anti-FcγRI antibodyattenuated peripheral nerve injury-induced neuropathic pain (**a, b**), and related to changes in expression of inflammatory mediators (**c-e**) in rats, related to inflammatory responses in cells (**f-h**). **a** The mechanical allodynia of neuropathic pain was induced by peripheral nerve injury, and significantly reversed by the anti-FcγRI antibody in rats. Each value represented the paw withdrawal threshold by von Frey test (*n* = 16). **b** The thermal allodynia was induced by peripheral nerve injury in rats, and remarkably attenuated by the anti-FcγRI antibody. Each value represented paw withdrawal thermal latency by heat tests (*n* = 16). **c-e** The expression of neuropeptide substance P (**c**) and cytokines TNF-α (**d**) and C_3_ (**e**) in the spinal cords were determined with real-time PCR (*n* = 3). **f-i** Modulation of FcγRI through anti-FcγRI antibody and IgG immune complex and effects on inflammatory responses in PC12 cells. The expression of FcγRI protein was determined and quantified by Western blotting analysis (*n* = 3). The protein levels were quantified by ImageJ software (**f**). Quantification of levels of TNF-α (**g**) and substance P (**h**) in PC12 cells incubated with IgG immune complex and/or the anti-FcγRI antibody using ELISA (*n* = 5). Data was presented as means ± S.E.M. *P < 0.05, compared to the C group; #*P* < 0.05, compared with the IC group; $$*P* < 0.01, compared with the S group; &*P* < 0.05, &&*P* < 0.01, &&&*P* < 0.001, compared with the NP group. Abbreviations: C, the control group (cells without IgG immune complex); IC, cells with IgG immune complex; IF, cells with IgG immune complex and the anti-FcγRI antibody; F, cells with the anti-FcγRI antibody; S, the sham group; NP, neuropathic pain model with pain behaviors; NF, neuropathic pain model with the anti-FcγRI antibody

First, we observed that the mechanical and thermal allodynia of neuropathic pain was induced by peripheral nerve injury in rats and lasted for 3 weeks (Fig. 1a). The detailed methods used in this study were described in the Additional file 1. Interestingly, the mechanical hyperalgesia was significantly attenuated after treatment with the anti-FcγRI antibody (4 μg/ml) in neuropathic pain rats on postoperative 3, 7, and 14 d, compared with the NP group (Fig. 1a). In the thermal behavioral tests, the latency was significantly extended by the anti-FcγRI antibody in neuropathic pain rats on postoperative 7, 14 and 21d, compared with that in the NP group (Fig. 1b). Because peripheral nervous injury leads to an increase of inflammatory mediators, linked to hyperalgesia and other pain behavioral changes [2], we anticipated that modulation of FcγRI will result in mediators changes in our pain model animals. We therefore investigated the influences of FcγRI on inflammatory mediators in the spinal cord of our neuropathic pain modelanimals. Indeed, we found that the levels of substance P, C_3_, and TNF-α were significantly higher in the NP group animals than those in the sham animals, as expected and supported by previous studies [15]. The anti-FcγRI antibody remarkably decreased the expression of these inflammatory mediators and neuropeptide in the neuropathic pain animals (Fig. 1c-e).

We next carried out cell-based studies to further investigate our animal-based findings. Specifically, the embryonically rat neural crest-originated PC12 cells were utilized and subjected to experiments of the following four groups: the control group (C); the IgG immune complex alone (IC, 0.1 μg/ml); and the group combined with IgG immune complex and anti-FcγRI antibody (IF, IgG immune complex 0.1 μg/ml and the anti-FcγRI antibody 0.2 μg/ml) and anti-FcγRI antibody alone (F, 0.2 μg/mL). We showed that the IgG immune complex significantly up-regulated FcγRI protein level compared to the control by Western blotting analysis (Fig. 1f). Additionally, the group combined with IgG immune complex and anti-FcγRI antibody significantly decreased FcγRI protein levels compared to the IgG immune complex alone (Fig. 1f). There were no significant changes comparing the anti-FcγRI antibody alone to the control. In addition, the IgG immune complex increased the expression of substance P and TNF-α compared to the control, which was attenuated by the addition of anti-FcγRI antibody (Fig. 1g-h). Our results from the animals and PC12 cells suggested that other various cell and animal models should be used in future studies to elucidate the mechanisms by which FcγRI modulates neuropathic pain. Collectively, these results suggested that FcγRI was involved in the IgG immune complex-induced inflammatory responses in cell-based studies, supporting our animal-based results.

Despite increased understanding of FcγRI underlying the pathophysiology of the neuroimmune system, its effects in neuropathic pain began to be elucidated. We utilized both animal and cell models in this study and showed that FcγRI is a critical player for the peripheral nerve injury-induced neuropathic pain. Importantly, the anti-FcγRI antibody attenuated pain-related neurobehavioral defects, and normalized changes of inflammatory cytokines. In summary, our studies showed that FcγRI modulates peripheral nerve injury-induced neuropathic pain in animals through regulating inflammatory mediators. Our finding may provide both novel knowledge of the pathogenesis underlyingthe neuropathic pain and may suggest new therapeutic strategies to alleviate pain.

## Supplementary information

**Additional file 1:** Materials and Methods, Supplementary Figures and Tables

## Data Availability

Available by contacting the corresponding author for reasonable request.

## Abbreviations

g: gram
NP: Neuropathic pain
SP: Substance P
