# Supplementary material for: The high-affinity IgG receptor FcγRI modulates peripheral nerve injury-induced neuropathic pain in rats
Source: Mol Brain. 2019 Oct 22;12:83. doi: 10.1186/s13041-019-0499-3 (PMC6805563; doi:10.1186/s13041-019-0499-3)
Supplement: Supplementary file 1 — Additional file 1: Materials and Methods, Supplementary Figures and Tables [file 13041_2019_499_MOESM1_ESM.docx]

Additional file

**Materials and methods**

**Animals**

All the animal procedures were performed in compliance with the National Institutes of Health Animal Care Guidelines. Adult male Sprague-Dawley rats (weight 150-200 g, 6 weeks old) were ordered from the Laboratory Animal Center of Shandong Lukang Pharmaceutical Co., Ltd (China). The rats were housed in separate cages at 24 ± 1°C and 50-60 % humidity with a 12/12-h light/dark cycle and had free access to standard laboratory water and food. The rats were divided into the following groups: sham (S; only separation of the sciatic nerve); NP (neuropathic pain; CCI model animals with pain); NF (NP + anti-FcγRI; CCI model animals treated with the anti-FcγRI antibody).

**IgG immune complex**

The IgG immune complex was synthesized by an antigen-antibody reaction and was used to active FcγRI. The antigen was a normal mouse IgG (Santa Cruz Biotechnology, CA), and the antibody was an affinity-purified rat anti-mouse IgG (Jackson ImmunoResearch, PA, USA). We replaced the IgG storage buffer with HEPES buffer using a Zeba™ spin desalting column (Thermo Scientific, Rockford, IL) to prevent possible sodium azide toxicity of the storage solution and to avoid non-specific reactions. Next, we synthesized IgG immune complex by incubating the antigen and antibody at a concentration of 40 μg/ml at a ratio of 1:1 at 25 °C for 1 h [[1-3](#_ENREF_1)].

**Chronic constriction injury model**

The surgical procedures were performed under aseptic condition, and anesthesia was performed with sodium pentobarbital (40 mg/kg, intraperitoneal) as previously reported [[4](#_ENREF_4)]. Briefly, the right sciatic nerve was isolated at the level of the midthigh and loosely ligated at 1-mm intervals using four chromic gut ties. The muscles and skin were layered and closed. In the sham group, the right sciatic nerve was isolated without ligation. The rats were tested, and those that demonstrated vigorous mechanical and thermal hypersensitivity were used for further experiments. For peri-sciatic injections injections, catheters were implanted into the rats according to methods previously described [[5](#_ENREF_5)]. Briefly, a gelatin sponge was cut into a block shape of 10 mm long, 5 mm wide, and 8 mm high. A PE-10 tube (15 cm) was inserted into one end of the gelatin sponge and was fixed to the gelatin sponge by wire. The prepared gelatin sponge was fixed on the right sciatic nerve that had been separated. The catheter was fixed on the surrounding muscles and sutured layer by layer. Then anti-FcγRI antibody (200 µl) was injected through the PE-10 tube using a micro-applicator twice a day for 3 days.

**Behavioral testing**

The paw withdrawal threshold (PWT) for mechanical stimulation was used to detect mechanical allodynia with an electronic von Frey apparatus as previously reported [[6](#_ENREF_6), [7](#_ENREF_7)]. The rats were placed into the test chamber for 1-hour habituation. Von Frey filaments were used to stimulate the surface of the hind paw. Briefly, a Frey tip of uniform diameter was attached to an electronic probe, and a readout displayed the force/weight at which the rodent retracted from the tip. The interval between consecutive stimulations was 5 min. When mechanical stimulation occurred, the rapid withdrawal or licking of the hind paw was considered a positive response. The mean of 3 measurements was calculated to obtain the withdrawal threshold for each rat. Thermal hyperalgesia was evaluated using the thermal withdrawal latency (TWL). Thermal sensitivity was assessed by 7370 plantar tests (Ugo Basile, Varese, Italy) [[7](#_ENREF_7), [8](#_ENREF_8)]. In brief, a source of thermal radiation was focused on the surface of the rat's hind paw. The thermal stimulation time was set to 25 s, and the heat source was turned off to prevent tissue damage. The average of 3 measurements was used for the final test results. The interval was > 5 min between consecutive trials. The experimenters conducting the behavioral tests were blinded to the treatments.

**Western blotting**

Proteins were analyzed by Western blotting according to previously reported methods [[9](#_ENREF_9)]. Briefly, the cells were homogenized with RIPA lysate buffer containing protease inhibitor cocktail (Sigma, USA) and centrifuged (13,000 × rpm, 4°C for 20 min) to collect the supernatant. The concentrations were detected using a bicinchoninic acid assay kit (Cwbio, China). The proteins were separated by 12% gel electrophoresis and then transferred onto polyvinylidene difluoride membranes (Millipore, USA). The blots were blocked with 5% w/v nonfat dry milk in TBST for 1 h and then incubated with primary antibodies, including the FcγRI antibody (1:200, Santa Cruz Biotechnology, CA) and the β-actin antibody (1:500; ZSbio, China), with gentle shaking at 4°C overnight. Next, the membranes were incubated with a secondary antibody (1:5000 or 1:8000, Cell Signaling Technology, USA) for 1 h at room temperature and then detected by chemiluminescence reaction.

**Enzyme-linked immunosorbent assay (ELISA)**

The levels of TNF-α (Boster, China) and substance P (Boster, China) were analyzed with the ELISA kits according to the manufacturer’s instructions. In brief, the medium was harvested after incubated with IgG immune complex and/or the anti- FcγRI antibody for 24 hours. All reagents were allowed to reach room temperature before use. The standard and sample (100 µl) were prepared and transferred to well and incubated for 90 min at 37 ̊. The plates were washed 5 times, and added 100 µl detection antibody, and incubated for 60 min at 37 ̊. Then the plates were washed 5 times and added 100 µl of chromogenic substrate to each well for 10 min, and then the stop solution (100 µl) was added. The plates were evaluated within 30 minutes of stopping the reaction. The optical density (O.D.) values were measured using a microplate reader (Bio-Rad, Japan) at 450 nm and 630 nm wavelengths.

**Real-time PCR**

Tissue was harvested at 7 d after CCI. Total RNA from the spinal cord was isolated with TRIzol reagent (Invitrogen, USA). The levels of substance P, C3 and TNF-α transcripts in the spinal cord was measured using SYBR Premix Ex Taq (TaKaRa, China) containing Ex Taq, SYBR Green I, Mg^2+^ and a dNTP mixture. The following genes were measured: substance P (forward: CGGAGCCCTTTGAGCATCTT; reverse: AGCATCCCGTTTGCCCATTA); C3 (forward: CAGGATGCCAAGAGTTCTATGA; reverse: GGTCGTTTGTGTCTGGAATAAAG); and TNF-α (forward: GCCGATTTGCCATTTCATAC; reverse: TGGAAGACTCCTCCCAGGTA). β-actin (forward: GCACTCTTCCAGCCTTCCTT; reverse: ACAGGTCTTTGCGGATGTCC); An ABI PRISM 7500 system was used for the following reactions: denaturation (94°C for 1 min); amplification and quantification (a total of 40 cycles; 95°C for 10 s, 58°Cfor 10 s, 72°Cfor 10 s with a single fluorescence measurement) and dissociation [[10](#_ENREF_10)].

**Cell culture**

The embryonically rat neural crest-originated PC12 cells were maintained in DMEM containing 10% fetal calf serum and 1% penicillin-streptomycin and have been previously reported[[11](#_ENREF_11)]. The cells were plated in plates at least 24hours before treatment with pharmacological substances. The cells were treated with the IgG immune complex (0.1 μg/mL) and/or the anti-FcγRI antibody (0.2 μg/mL) for 24 hours[[2](#_ENREF_2)].

**Statistical analysis**

All data are expressed as the mean ± S.E.M.. Using treatment and time as the main factors, a two-way ANOVA followed by Bonferroni post hoc test was used to analyze the behavioral data. The methods for analyzing proteins and mRNA levels were described in the figures and tables below. Statistical tests were performed with GraphPad Prime 5.02, and P < 0.05 was considered statistically significant.

**
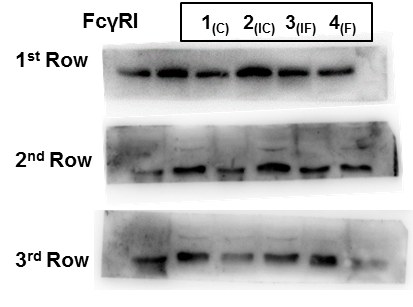

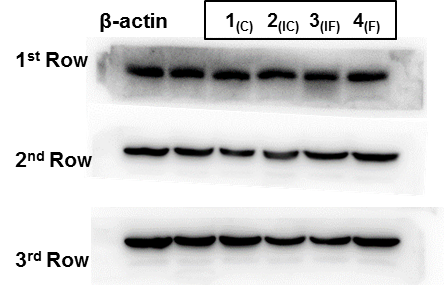
Supplementary Figures and Tables**

**Figure S1**: Protein analysis of samples from cells treated with C (control), IC (the IgG immune complex), IF (the IgG immune complex with the anti- FcγRI antibody) and F (the anti- FcγRI antibody). The membranes were probed and developed to detect FcγRI (left) and β-actin (right), respectively. For quantification, the FcγRI levels were normalized to β-actin for each sample to account for loading differences.

| 1^st^ Row | C | IC | IF | F |
| --- | --- | --- | --- | --- |
| FcγRI | 22532.34 | 39644.29 | 23897.83 | 23202.53 |
| β-actin | 24052.91 | 25509.98 | 23597.98 | 23989.37 |
| FcγRI/β-actin | 0.936782 | 1.55407 | 1.012707 | 0.967201 |

| 2^nd^ Row | C | IC | IF | F |
| --- | --- | --- | --- | --- |
| FcγRI | 10522.09 | 26884.94 | 15096.23 | 14977.36 |
| β-actin | 18154.77 | 17830.89 | 22757.84 | 22882.33 |
| FcγRI/β-actin | 0.579577 | 1.507773 | 0.663342 | 0.654538 |

| 3^rd^ Row | C | IC | IF | F |
| --- | --- | --- | --- | --- |
| FcγRI | 16263.09 | 29736.25 | 25043.3 | 11563.55 |
| β-actin | 30429.46 | 25559.51 | 25322.32 | 26820.64 |
| FcγRI/β-actin | 0.534452 | 1.163412 | 0.988981 | 0.431144 |

| Mean ± SEM | C | IC | IF | F | F value | P value |
| --- | --- | --- | --- | --- | --- | --- |
| FcγRI/β-actin | 0.684±0.127 | 1.408±0.123 | 0.888±0.113 | 0.684±0.156 | 6.856 | 0.0133 |

**Table S1**: Quantified numerical data for samples from cells treated with C (control), IC (the IgG immune complex), IF (the IgG immune complex with the anti- FcγRI antibody) and F (the anti- FcγRI antibody). The levels of FcγRI from C, IC, IF and F were 0.684±0.127, 1.408±0.123, 0.888±0.113 and 0.684±0.156, respectively. The data was analyzed by one-way ANOVA followed by student’s t-test for two group comparisons.

| Group | S | NP | NF | F value | P value |
| --- | --- | --- | --- | --- | --- |
| SP | 1.001±0.034 | 1.313±0.108 | 0.629±0.052 | 59.56 | 0.0001 |
| TNFα | 86.24±3.26 | 121.56±7.89 | 98.44±4.21 | 16.31 | 0.0037 |
| C3 | 27.84±0.95 | 49.30±4.06 | 39.96±1.54 | 25.54 | 0.0012 |

**Table S2**. Data of mRNA levels of substance P and cytokines TNFα and C3 in the spinal cord of neuropathic pain rat with anti-FcγRI antibody. The data were analyzed by one-way ANOVA followed by Tukey’s post hoc test.

| Group | C | IC | IF | F | F value | P value |
| --- | --- | --- | --- | --- | --- | --- |
| SP | 86.24±3.26 | 121.56±7.89 | 98.44±4.21 | 88.13±3.69 | 10.10 | 0.0006 |
| TNFα | 27.84±0.95 | 49.30±4.06 | 39.96±1.54 | 25.86±2.35 | 19.08 | <0.0001 |

**Table S3**. Data of substance P, and TNFα protein levels in the PC12 cells treated with IgG immune complex and anti-FcγRI antibody. The data were analyzed by one-way ANOVA followed by Tukey’s post hoc test.

**References**

1. Andoh T, Kuraishi Y: **Direct action of immunoglobulin G on primary sensory neurons through Fc gamma receptor I**. *FASEB journal : official publication of the Federation of American Societies for Experimental Biology* 2004, **18**(1):182-184.

2. Qu L, Zhang P, LaMotte RH, Ma C: **Neuronal Fc-gamma receptor I mediated excitatory effects of IgG immune complex on rat dorsal root ganglion neurons**. *Brain, behavior, and immunity* 2011, **25**(7):1399-1407.

3. Qu L, Li Y, Pan X, Zhang P, LaMotte RH, Ma C: **Transient receptor potential canonical 3 (TRPC3) is required for IgG immune complex-induced excitation of the rat dorsal root ganglion neurons**. *The Journal of neuroscience : the official journal of the Society for Neuroscience* 2012, **32**(28):9554-9562.

4. Chacur M, Milligan ED, Gazda LS, Armstrong C, Wang H, Tracey KJ, Maier SF, Watkins LR: **A new model of sciatic inflammatory neuritis (SIN): induction of unilateral and bilateral mechanical allodynia following acute unilateral peri-sciatic immune activation in rats**. *Pain* 2001, **94**(3):231-244.

5. Wei XH, Zang Y, Wu CY, Xu JT, Xin WJ, Liu XG: **Peri-sciatic administration of recombinant rat TNF-alpha induces mechanical allodynia via upregulation of TNF-alpha in dorsal root ganglia and in spinal dorsal horn: the role of NF-kappa B pathway**. *Experimental neurology* 2007, **205**(2):471-484.

6. Chaplan SR, Bach FW, Pogrel JW, Chung JM, Yaksh TL: **Quantitative assessment of tactile allodynia in the rat paw**. *Journal of neuroscience methods* 1994, **53**(1):55-63.

7. Zhang C, Ward J, Dauch JR, Tanzi RE, Cheng HT: **Cytokine-mediated inflammation mediates painful neuropathy from metabolic syndrome**. *PloS one* 2018, **13**(2):e0192333.

8. Hargreaves K, Dubner R, Brown F, Flores C, Joris J: **A new and sensitive method for measuring thermal nociception in cutaneous hyperalgesia**. *Pain* 1988, **32**(1):77-88.

9. Liang Y, Jiang W, Zhang Z, Yu J, Tao L, Zhao S: **Behavioral and morphological evidence for the involvement of glial cells in the antinociceptive effect of najanalgesin in a rat neuropathic pain model**. *Biological & pharmaceutical bulletin* 2012, **35**(6):850-854.

10. Abdelsamie SA, Li Y, Huang Y, Lee MH, Klein RL, Virella G, Lopes-Virella MF: **Oxidized LDL immune complexes stimulate collagen IV production in mesangial cells via Fc gamma receptors I and III**. *Clin Immunol* 2011, **139**(3):258-266.

11. Chen Y, Liang Y, Zhang Y: **[FcgammaRIis involved in lipopolysaccharide-induced apoptosis of PC12 cells]**. *Xi bao yu fen zi mian yi xue za zhi = Chinese journal of cellular and molecular immunology* 2017, **33**(6):746-750.
